# The Reliability of the Microsoft Kinect and Ambulatory Sensor-Based Motion Tracking Devices to Measure Shoulder Range-of-Motion: A Systematic Review and Meta-Analysis

**DOI:** 10.3390/s21248186

**Published:** 2021-12-08

**Authors:** Peter Beshara, David B. Anderson, Matthew Pelletier, William R. Walsh

**Affiliations:** 1 Department of Physiotherapy, Prince of Wales Hospital, Sydney, NSW 2031, Australia; 2 Prince of Wales Clinical School, Faculty of Medicine, University of New South Wales, Sydney, NSW 2031, Australia; m.pelletier@unsw.edu.au (M.P.); w.walsh@unsw.edu.au (W.R.W.); 3 Surgical & Orthopaedic Research Laboratories, Prince of Wales Hospital, Sydney, NSW 2031, Australia; 4 School of Health Sciences, Faculty of Medicine and Health, The University of Sydney, Sydney, NSW 2006, Australia; david.anderson1@sydney.edu.au

**Keywords:** Kinect, reliability, range of motion, inertial sensor, meta-analysis

## Abstract

Advancements in motion sensing technology can potentially allow clinicians to make more accurate range-of-motion (ROM) measurements and informed decisions regarding patient management. The aim of this study was to systematically review and appraise the literature on the reliability of the Kinect, inertial sensors, smartphone applications and digital inclinometers/goniometers to measure shoulder ROM. Eleven databases were screened (MEDLINE, EMBASE, EMCARE, CINAHL, SPORTSDiscus, Compendex, IEEE Xplore, Web of Science, Proquest Science and Technology, Scopus, and PubMed). The methodological quality of the studies was assessed using the consensus-based standards for the selection of health Measurement Instruments (COSMIN) checklist. Reliability assessment used intra-class correlation coefficients (ICCs) and the criteria from Swinkels et al. (2005). Thirty-two studies were included. A total of 24 studies scored “adequate” and 2 scored “very good” for the reliability standards. Only one study scored “very good” and just over half of the studies (18/32) scored “adequate” for the measurement error standards. Good intra-rater reliability (ICC > 0.85) and inter-rater reliability (ICC > 0.80) was demonstrated with the Kinect, smartphone applications and digital inclinometers. Overall, the Kinect and ambulatory sensor-based human motion tracking devices demonstrate moderate–good levels of intra- and inter-rater reliability to measure shoulder ROM. Future reliability studies should focus on improving study design with larger sample sizes and recommended time intervals between repeated measurements.

## 1. Introduction

The clinical examination of individuals with shoulder pathology routinely involves the measurement of range-of-motion (ROM) to diagnose, evaluate treatment, and assess disease progression [1,2,3]. The shoulder complex involves the coordination of the acromioclavicular, glenohumeral and scapulothoracic joints, to allow motion in three biomechanical planes, specifically the sagittal, coronal, and axial planes [4]. Forward flexion and elevation occur in the sagittal plane; abduction and adduction occur in the coronal plane; and internal and external rotation occur along the long axis of the humerus [5].

The shoulder joint’s complex multiplanar motion presents a challenge for clinicians to accurately measure ROM and upper limb kinematics [6,7]. Prior attempts to implement a global coordinate system to describe shoulder movement and define arm positions in space [8] have failed to gain clinical consensus due to practical difficulties. The biomechanical complexity of the shoulder is demonstrated by the synergy of movements necessary for a person to perform activities of daily living. Activities such as reaching for a high shelf or hair washing requires a combination of flexion and adduction. Similarly, reaching behind the back pocket requires a combination of internal rotation, extension, and adduction. Although many models have been proposed in literature, it nevertheless remains difficult to determine the contribution of individual components of glenohumeral joint and scapulothoracic joint motions. Therefore, the reliability of any tools used for ROM measurement is important for clinicians to make informed decisions regarding patient management [9].

According to the American Academy of Orthopaedic Surgeons (AAOS), normal active ROM of the shoulder is 180° for flexion and abduction, and 90° for external rotation [10]. However, a number of age and health-related variables exist that can influence shoulder ROM, including factors such as gender, work history, and hand-dominance. Studies have demonstrated an overall reduction in ROM across all shoulder movements with age in subjects with no shoulder pathology [11,12]. Gill et al. [13] reported age-related decreases in right active shoulder flexion by 43° in males, and 40.6° in females, and right active shoulder abduction by 39.5° and 36.9°, respectively. Authors also noted a decline in external rotation range, particularly among females. The age-related causes of decreased ROM occur from sarcopenia (loss of muscle mass due to a decrease in Type-II fibres), changes in fat redistribution and slower collagen fibre reproduction, leading to reduced elasticity and shortened ligaments and tendons [14,15].

A goniometer is the most commonly used instrument by clinicians to measure joint position and ROM [16,17]. It is essentially a 360° protractor, comprising a stationary arm, a movable arm, and a fulcrum. When used correctly by a trained clinician, the benefits of goniometry’s low cost and portability [18,19] are offset by the disadvantages of low inter-reliability [20,21] and measurement variability between clinicians [22,23,24,25]. Furthermore, the two-handed requirement of using a goniometer makes it difficult to stabilise the trunk and scapula, resulting in an increased likelihood of measurement error [26]. Alternatively, inclinometers measure ROM relative to the line of gravity and demonstrate improved inter-rater reliability compared with goniometry when assessing shoulder measurements [27,28]. The drawbacks of inclinometery include higher cost, poorer accessibility, and possible technical errors due to misplacing body landmarks or not sustaining constant pressure with the device during movements.

With the increasing popularity, accessibility, and convenience of smartphones and similar devices [29], the potential exists for these electronics to become a clinician’s measuring tool of choice. Smartphones with inbuilt accelerometers and magnetometers can utilise inclinometer or sensor-based applications to calculate shoulder joint angles [30,31,32]. Similarly, digital inclinometers or goniometers are compact, portable, and lightweight. However, a degree of training is required for the clinician to accurately determine a zero point and limit measurement error [33]. Although more costly than traditional manual methods, digital inclinometers and smartphones eliminate the need for realignment and require only one hand to operate [34]. Additionally, the ability to transmit measurements may decrease transpositional or other manual entry errors.

Further advancements in technology over the last decade have led researchers to adopt hands-free motion sensing input devices to estimate human joint ROM [35]. The Microsoft Kinect sensor was originally developed as an add-on for the Xbox 360 gaming console (Microsoft Corp., Redmond, WA, USA) [36] and has since been modified for application in real-world settings including telehealth [37,38], education [39,40] and kinematic motion analysis [41,42,43]. The Kinect sensor combines a regular colour camera with a depth camera that comprises an infrared laser projector and infrared camera. The Windows Software Development Kit (SDK) 2.0 has allowed for the creation of applications that utilise the Kinect’s gesture recognition capability to implement joint orientation and skeletal tracking for 25 joint positions in the standing or seated positions [44,45]. Given its potential breadth of use, the Kinect is emerging as a promising clinical tool for kinematic analysis by virtue of its function as a markerless system to estimate the 3-D positions of several body joints [46].

Ambulatory sensor-based human motion tracking devices such as inertial measurement units (IMUs) comprise accelerometers, gyroscopes, and magnetometers. IMUs measure linear acceleration and angular velocities, combining data to obtain a 3-D position and orientation of a body [47]. The miniaturisation, wearability, and low cost of IMUs over the last decade has made it a desirable alternative to expensive motion-capturing systems for measuring joint angles [48,49,50]. Prior studies evaluating the precision of IMUs have reported mean average errors of <5.0° for upper limb measurements [51,52,53]. However, IMU accuracy varies depending on the amount of ROM a joint can produce [53], type of device [54], and fusion algorithm used [55].

Prior to using any device for ROM assessment in clinical practice, it is important to establish the measurement properties of validity and reliability [56]. Several studies have previously validated the Microsoft Kinect [57,58,59,60], IMUs [61,62,63], digital inclinometers [64,65,66,67] and smartphone applications [68,69,70] against a prescribed “gold standard” for shoulder ROM measurement.

For the purposes of this review, authors examined reliability, reflecting the degree to which measurements are consistent over time and across different observers or raters [71]. The two recognised types of reliability in the literature are: intra-rater reliability—the amount of agreement between repeated measurements of the same joint position or ROM by a single rater, and inter-rater reliability—the amount of agreement between repeated measurements of the same joint position or ROM by multiple raters [72].

Absolute reliability is considered equally important and indicates the amount of variability for repeated measurements between individuals [73,74]. Examples include the standard error of measurement (SEM), coefficient of variation (CV), and Bland and Altman’s 95% limits of agreement [74]. Absolute measures of reliability allow clinicians to evaluate the level of measurement error and determine whether any changes in ROM signify a real change in their patients [75,76].

Few studies have summarised or appraised the literature on the reliability of the Microsoft Kinect, IMUs, smartphones, and digital inclinometers for human joint ROM measurement. Previous systematic reviews have focused on applying the Kinect for stroke rehabilitation [77,78,79], and Parkinson’s disease [80,81,82]. Only one systematic review on reliability was identified, which examined using the Kinect to assess transitional movement and balance [83]. To our knowledge, no systematic appraisals of studies on the intra- and inter-rater reliability of the Kinect and ambulatory sensor-based motion tracking devices for shoulder ROM measurement have been conducted.

Therefore, the aim of this article is to review systematically, and appraise critically, the literature investigating the reliability of the Kinect and ambulatory sensor-based motion tracking devices for measuring shoulder ROM.

The specific study questions for this systematic review were:

1. What is the intra- and inter-rater reliability of using the Microsoft Kinect, inertial sensors, smartphone applications, and digital inclinometers to calculate a joint angle in the shoulder?
2. What are the types of inertial sensors, smartphone applications, and digital inclinometers currently used to calculate a joint angle in the shoulder?
3. What clinical populations are utilising motion-tracking technology to calculate the joint angle in the shoulder?
4. Which anatomical landmarks are used to assist the calculation of joint angle in the shoulder?

## 2. Materials and Methods

The protocol for this review was devised in accordance with the Preferred Reporting Items for Systematic Reviews and Meta-Analyses (PRISMA) statement guidelines [84], published with PROSPERO on the 8 December 2017 (CRD 42017081870).

The search strategy was developed and refined by previous systematic reviews investigating reliability [85,86]. A database search of Medline (via OvidSP), EMBASE (via OvidSP), EMCARE (via Elsevier), CINAHL (via Ebsco), SPORTSDiscus (via Ebsco), Compendex (via Engineering Village), IEEE Xplore (via IEEE), Web of Science (via Thomson Reuters), Proquest Science and Technology (via Proquest), Scopus (via Elsevier), and Pubmed was initially performed on 30 January 2020 by two independent reviewers (PB, DB). These databases were searched from their earliest records to 2020. An updated search was completed on 17 December 2020. Details of the search strategy are found in Supplementary S1. The reference lists of all included studies were screened manually for additional papers that met the a priori inclusion criteria.

### 2.1. Inclusion and Exclusion Criteria

Studies were included if they met the following criteria: published in peer-reviewed journals; measured human participants of all ages; used the Microsoft Kinect, inertial sensors, smartphone applications, or digital inclinometers to measure joint ROM of the shoulder joint and assessed the intra- and/or inter-rater reliability of these devices; published in English and had full text available. Case studies, abstracts only and “grey” literature was not included. Studies only investigating validity, scapular or functional shoulder movements were excluded, as the aim of the review was to examine the reliability of specific shoulder joint movements commonly measured in clinical practice.

The titles and abstracts of studies were retrieved using the search strategy (Supplementary S1) and screened independently by two review authors (PB, DB). Full text versions that met the selection criteria were uploaded to an online systematic review program (Covidence) for independent review by both reviewers (PB, DB). Any disagreements on eligibility were initially resolved by discussion between reviewers and resolved by a third reviewer (WRW), if necessary.

### 2.2. Data Extraction

A standardised, pre-piloted form was used to extract data from the included studies for assessment of study quality and evidence synthesis. The following information was extracted for each study: bibliometric (author, title, year of publication, funding sources); study methods (study design, country, setting, description and number of raters, type of shoulder joint movements; type of movement (active ROM (aROM) or passive (pROM)); number of sessions, session interval, type and description of technology); participants (recruitment source, number of drop outs, sample size, age, gender inclusion criteria); anatomical landmarks, statistical methods (type of reliability), and outcomes (intraclass correlation coefficient (ICC) values).

### 2.3. Evaluation of Reliability Results

Reliability was assessed using ICCs; an ICC value approaching 1 was indicative of higher reliability. The level of intra- and inter-rater reliability was determined by the criteria identified by Swinkels et al. [87]. Intra-rater reliability was considered good with an ICC > 0.85, moderate with ICCs 0.65–0.85, and poor with an ICC < 0.65. Inter-rater reliability was considered good with an ICC > 0.80, moderate with ICCs 0.60–0.80, and poor with an ICC < 0.60.

### 2.4. Evaluation of the Methodological Quality of the Studies

The two review authors independently assessed the methodological quality of each included paper using the latest (2020) Consensus-based Standards for the selection of health Measurement Instruments (COSMIN) Risk of Bias tool [88]. The studies were rated against a specific set of criteria, with nine items assessing reliability standards and eight items assessing measurement error standards. To satisfy item seven of the measurement error standards, the study had to report absolute reliability statistics (standard error of measurement (SEM), smallest detectable change (SDC) or Limits of Agreement (LOA)). Each item was graded on a four-point scale as either very good, adequate, doubtful, or inadequate. The worst-score-count method was applied in accordance with the COSMIN protocol; the overall score was determined by the lowest score awarded for the measurement property, as used in previous studies [89,90].

### 2.5. Data Analysis

Meta-analyses of relative intra- and inter-rater reliability were performed for studies with outcome measures that reported comparable data. Pooled analysis was completed for maximal aROM and pROM. The right-hand dominant value for the healthy, asymptomatic population was included for analysis. Studies with multiple reliability values were pooled and one overall mean result was reported. If a single study reported values for more than one rater, the mean value was reported. Reflecting clinical practice, any reliability values taken in supine position were included in the pROM analysis, and the standing or sitting positions were included in the aROM analysis.

## 3. Results

### 3.1. Flow of Studies

**Figure 1:**
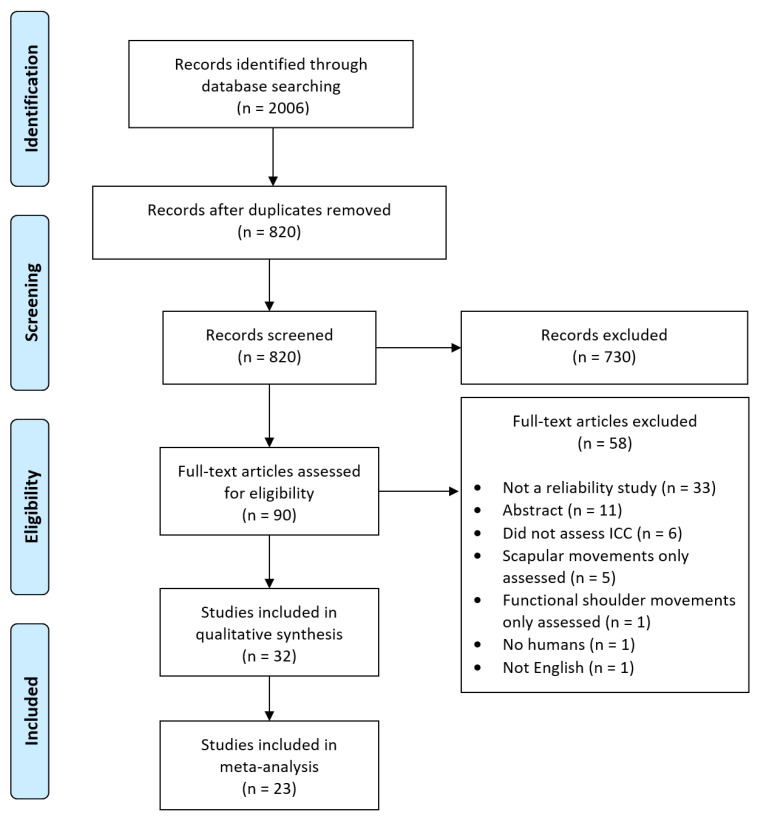
Flow chart of the systematic review process.

A flowchart of the different stages of the article selection process is outlined in Figure 1. From the 2006 studies identified, 32 studies [91,92,93,94,95,96,97,98,99,100,101,102,103,104,105,106,107,108,109,110,111,112,113,114,115,116,117,118,119,120,121,122] were found to meet the criteria for inclusion. In total, nine studies reported reliability for the Microsoft Kinect; six studies for wearable inertial sensors; seven studies for smartphone/mobile applications; and ten studies for digital inclinometers or goniometers.

### 3.2. Description of Studies

The characteristics of the included studies are summarised in Table 1. A total of 1117 participants were included in this review, with a mean age ranging from 17.0 to 56.1 years of age. The mean sample size was 35 participants with a considerable range (minimum, 1; maximum 155) and variance (SD, 32.1). Six studies recruited more than 50 participants and five studies recruited fewer than ten participants. In 13 of the studies, there was a higher percentage of women compared to men. Most studies (n = 26) recruited participants who were healthy and asymptomatic. Participants with shoulder pain or pathology were reported in six studies.

A physical therapist (PT) was the most reported type of rater (n = 12 studies). In six studies the rater was a medical practitioner (MP), and in two studies a PT student was the sole primary rater. Thirteen studies did not report the profession of the rater.

The shoulder movements assessed across all studies included flexion, extension, abduction, external rotation, internal rotation, and scaption. A total of 24 studies only assessed aROM; eight studies assessed pROM, and two assessed both. The most common measuring position was standing (n = 10 studies), followed by seated (n = 6 studies) and supine (n = 3 studies). There were twelve studies that used a combination of supine and standing, side-lying, prone or seated positions. Only one study did not report the position used.

The majority of studies (n = 25) reported two sessions; five studies had one session, and two studies involved three sessions. The time interval between assessments varied considerably from 10 s to 7 days. The most common consecutive measurements were on the same day (n = 13) followed by 7 days (n = 5).

### 3.3. Intra and Inter-Rater Reliability

Results for intra- and inter-rater reliability are shown in Table 2. The last column of Table 2 indicates the level of reliability, grouped by type of device, and includes the shoulder movement assessed.

#### 3.3.1. The Microsoft Kinect

Six studies assessed intra-rater reliability [93,94,99,105,106,107], one study assessed inter-rater reliability [112] and another study assessed both [101]. Two studies reported good intra-rater reliability (ICC > 0.85) for all shoulder movements [105,107]. The remaining four studies reported varying levels of intra-rater reliability, ranging from poor (ICC < 0.65), moderate (ICC 0.65–0.85) to good, dependent on the shoulder movements assessed. Shoulder external and internal rotation demonstrated moderate to good levels of intra-rater reliability [94,99,106]. Two studies reported good inter-rater reliability (ICC > 0.80) for shoulder flexion, extension, and abduction [101,112]. Intra-rater reliability for coupling inertial sensors with the Kinect was moderate to good for flexion, and poor to moderate for abduction [92].

#### 3.3.2. Inertial Sensors

One study assessed intra-rater reliability [114], three studies assessed inter-rater reliability [96,117,118] and two studies assessed both [100,102]. Three studies reported moderate to good intra-rater reliability using one, two or four wearable inertial sensors [100,102,114]. Inter-rater reliability was good or moderate in four studies for shoulder flexion, extension, abduction, external and internal rotation [96,102,117,118]. A wider range of poor (ICC < 0.60) to good inter-rater reliability was reported in two studies for shoulder abduction, external and internal rotation [100,102].

#### 3.3.3. Smartphone/Mobile Applications

A total of five of seven studies [95,110,113,116,120] assessed intra-rater and inter-rater reliability. All shoulder movements across most of the studies demonstrated moderate or good levels of intra- and inter-rater reliability. Only one study reported a wider range of reliability values, between poor and good, for flexion and scaption [116].

#### 3.3.4. Digital Inclinometer/Goniometer

Two studies assessed intra-rater reliability [104,121], one study assessed inter-rater reliability [103], and seven studies assessed both [91,97,98,108,109,115,119]. Intra-rater reliability was predominately moderate to good for all shoulder movements (n = 7). Two studies reported poor to moderate intra-rater reliability for external and internal rotation [91,104]. Poor inter-rater reliability was reported in five studies [91,103,108,115,119]. Only two studies reported good intra- and inter-rater reliability for all shoulder movements [97,109].

### 3.4. Methodological Evaluation of the Measurement Properties

Of the thirty-two included studies, only two [109,110] scored very good on all items of the COSMIN reliability standards checklist. A total of 24 studies scored adequate, five were rated doubtful and one was rated inadequate. Table 3 lists the COSMIN standards of reliability checklist and all subsequent scores.

Using the COSMIN criteria, only one study [109] was found to have a very good score on all items for the measurement error standards. A total of 18 studies scored adequate, with two rated doubtful and 11 rated inadequate. Table 4 lists all items of the COSMIN standards on measurement error checklist and the subsequent paper scores.

### 3.5. Synthesis of Results (Meta-Analysis)

**Figure 2:**
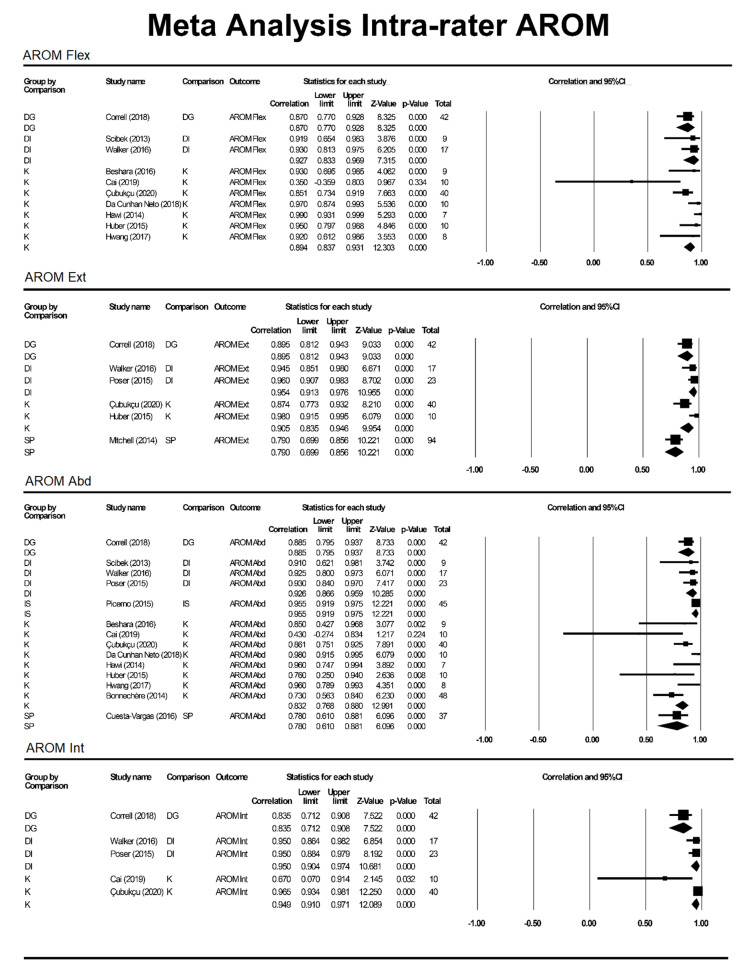
Meta-analysis for intra-rater AROM. DG = digital goniometer, DI = digital inclinometer, IS = inertial sensor, K = Kinect, SP = smartphone, Abd = abduction, AROM = active range of motion, Int = internal rotation, Ext = external rotation.

**Figure 3:**
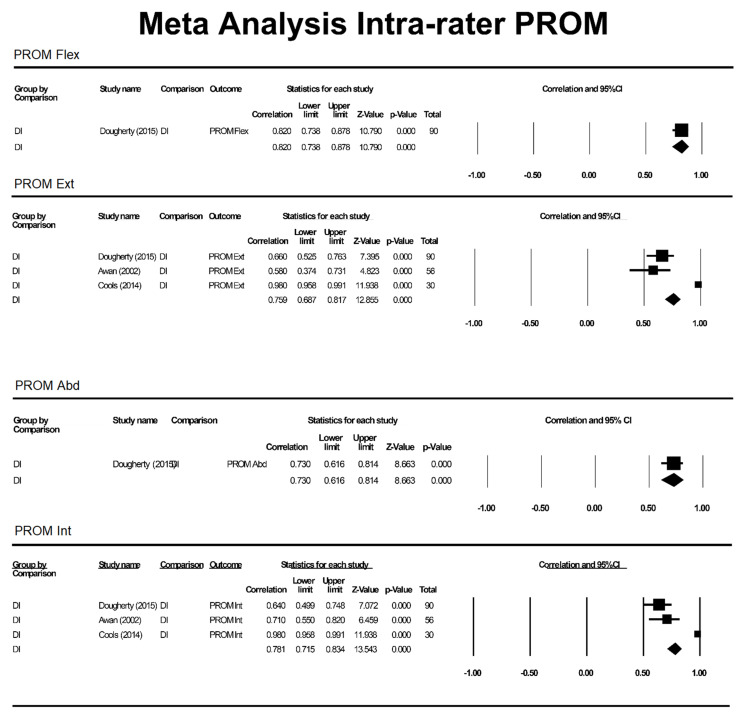
Meta-analysis for intra-rater pROM. DI = digital inclinometer, PROM = passive range of motion, Flex = flexion, Int = internal rotation, Ext = external rotation.

**Figure 4:**
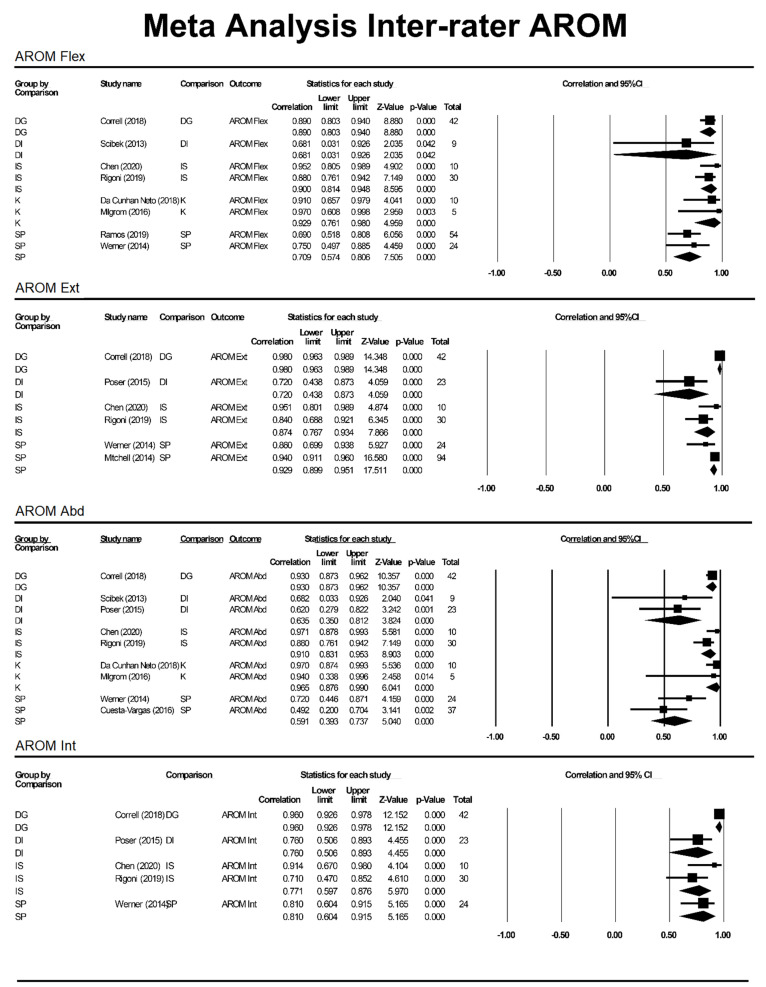
Meta-analysis for inter-rater aROM. DG = digital goniometer, DI = digital inclinometer, IS = inertial sensor, K = Kinect, SP = smartphone, Abd = abduction, Int = internal rotation.

ICC values were included from studies with n > 1 participant included in intra- and inter-rater reliability analysis. The ICC values for outcome measures (aROM or pROM for abduction, flexion, internal rotation, external rotation) were individually assessed based on motion and grouped by method (K, SP, DG, DI and IS) to produce a pooled correlation with a 95%CI (Figure 2, Figure 3 and Figure 4).

### 3.6. Anatomical Landmarks

Twenty-three studies identified the anatomical landmarks for each device and are summarised in Table 5. A total of six studies reported using a vector from the shoulder joint to the elbow for the Microsoft Kinect [92,93,94,105].

Five studies identified the anatomical landmarks for inertial sensor placement [100,102,114,117,118]. All studies used a sensor located on the upper arm that was either unspecified (n = 2), placed on the middle third of the humerus (n = 3), or attached 10 cm distal to the lateral epicondyle (n = 1). Two studies placed a sensor on the flat part of the sternum [100,102]. Only two studies reported using a lower arm sensor on the wrist [102,118].

Anatomical positions for smartphone device placement were described in five studies [95,110,111,113,120]. The most common attachment was on the humerus (n = 3) followed by positions at the wrist (n = 2).

Seven studies reported anatomical landmarks for digital inclinometers [98,104,108,109,115,119,121]. Locations were predominately determined by the type of shoulder movement performed, orientation, and assessment position.

## 4. Discussion

Thirty-two studies investigating four different types of devices were included in this review. A thorough search of relevant literature found no previous systematic review of intra-rater and inter-rater reliability of the Microsoft Kinect and ambulatory sensor-based motion tracking devices to measure shoulder ROM.

Good intra-rater reliability for multiple types of shoulder movement was demonstrated with the Kinect [105,107], smartphone applications [95], and digital inclinometers [97]. The Kinect consistently demonstrated higher intra-rater ICC values over other devices for all shoulder movements. Only one study reported poor intra-rater reliability for measuring shoulder extension with the Kinect [99]. Overall, inertial sensors, smartphones, and digital inclinometers demonstrated moderate to good intra-rater reliability across all shoulder movements.

Good inter-rater reliability for more than one type of shoulder movement was demonstrated with the Kinect [101,112], smartphone applications [95,111], and a digital inclinometer/goniometer [97,98]. Inertial sensors predominately exhibited moderate to good inter-rater reliability across all types of shoulder movements. Broader ranges of inter-rater reliability (between poor to moderate) were more commonly reported with digital goniometers.

### 4.1. Quality of Evidence

All included studies and measurement properties were assessed for their methodological quality using the COSMIN tool. The methodological quality ranged from doubtful to very good for reliability standards. The strict COSMIN criteria of using the worst-score counts to denote the overall score resulted in only two very good studies [109,110], which reported moderate or good reliability for using a digital inclinometer and a smartphone device. An adequate rating was scored by five studies for the Kinect, six studies for inertial sensors, five studies for smartphone applications, and eight studies for digital inclinometers/goniometers.

Five studies missed achieving an overall very good rating due to receiving only an adequate score for the time interval between measurements (COSMIN item two). The authors acknowledge an appropriate time interval depends on the stability of the construct (COSMIN item one), and the target population [88]. The time interval must be adequately distanced to avoid recall bias, yet within a compact enough window to distinguish genuine differences in measurements from clinical change [123,124,125]. Studies had a time interval ranging from the same day (22/32) to 7 days (4/32) between two repeated measurements. Ideal time intervals of 2–7 days have been recommended to minimise the risk of a learning effect, random error, or other modifying factors that can affect the movement pattern [126,127].

Small sample sizes contributed to five studies scoring doubtful or inadequate, in accordance with COSMIN item six. An insufficient sample size may not detect true differences and reduces the power of the study to draw conclusions [128]. Of the 32 included studies, a power analysis for sample size calculation was reported in only four (12.5%) studies [97,98,100,104]. The latest COSMIN checklist has removed the standards for adequate sample sizes, as the authors suggested that several small high-quality studies can together provide good evidence for the measurement property [129]. The guidelines recommend a more nuanced approach that considers several factors including the type of ICC model. Studies with small sample sizes were considered acceptable if the authors justified the reasons outlining its adequacy [129]. Therefore, for methodological quality, reviewers scored sample sizes of 1 inadequate, ˂10 doubtful, <30 adequate and ≥30 very good. This criterion was based on literature citing a rule of thumb of recruiting 19–30 participants when conducting a reliability study [130,131,132].

With respect to measurement error assessment, just over one-half of the studies (18/32) scored adequate, and one scored very good for methodological quality. Eleven studies were rated inadequate, as they failed to calculate SEM, SDC, LoA or CV values (COSMIN item seven). Two studies [92,107] were rated doubtful due to minor methodological flaws (COSMIN item six); notably, this strict item offered reviewers no adequate option.

Reliability and measurement error are inextricably linked, and a highly reliable measurement contains little measurement error. A clinician can confidently verify real changes in patient status if the measured change from the last measurement is larger than the error associated with the measurement [133]. The minimal detectable clinical difference at a 90% confidence level (MDC90) is the minimal value to determine whether a change has occurred [72]. MDC values are open to interpretation and are based on clinical judgement. For shoulder ROM measurement, differences between observers which exceed 10° are deemed unacceptable for clinical purposes [103].

The Kinect and inertial sensors demonstrated low SEM and MDC values for measuring most types of shoulder movements [96,99,102,106]. Similarly, for the Kinect, low CV values (1.6%, 5.9%) were reported for shoulder abduction [93]. Smartphones had moderate SEM and MDC values, with better (smaller) errors demonstrated for intra-rater analysis [118], abduction and forward flexion [122], and higher target angles [116]. One study comparing smartphone measurements with universal goniometry, analysed Bland–Altman plots to indicate narrow LoA and excellent agreement, particularly for glenohumeral abduction [111]. Digital inclinometers demonstrated low MDC90 values, ranging from ≥2.82° to 5.47° [97], 4° to 9° [108], and 5° to 12° [121] for inter-rater analysis. Four studies reported acceptable differences between observers of <±10° [97,103,108,121] for most inclinometer measurements.

### 4.2. Clinical Implications

The Microsoft Kinect is an affordable depth imaging technology that can conveniently and reliably measure shoulder aROM. As a low-cost markerless system, the Kinect can provide clinicians with fast, real-time objective data to quantify shoulder kinematics. The Kinect’s visual feedback can aid in patient motivation by way of monitoring treatment and disease progression. The massive amounts of kinematic data generated allows clinicians to potentially analyse shoulder motion paths and correlate specific movement patterns to shoulder pathology [94]. Moreover, higher clinical efficiency arises from relying less on time and labour-intensive patient-reported outcome measures. The portability of the Microsoft Kinect over expensive motion capture systems permits its practical use in private clinics, rehabilitation centres, and home settings [107].

All studies were limited to motion performed along the anatomical planes. The simplicity of calculating the angles between two corresponding vectors does not take into account movements that occur outside the plane. In contrast to goniometric measurements, Lee et al. [60] found subjects could abduct their shoulders to a greater degree in front of the Kinect because their movements were not controlled in a given anatomical plane by an examiner. The authors performed a supplementary experiment that compared goniometric and Kinect shoulder measurements in rapid succession within three cardinal planes. Results demonstrated a significant decrease in 95% limit of agreement between both methods in all directions. It was concluded that the variability was due to the unrestricted motion of the Kinect.

With respect to reliability, one study reported lower repeatability with the Kinect in the frontal and transverse planes compared to the sagittal plane [94]. Another study reported large discrepancies for precise shoulder angle measurements with the Kinect [106].

Discrepancies between standing, sitting, and lying positions can also be a source of difference for shoulder ROM measurements [134,135]. One study [60] reported discrepancies between goniometric shoulder ROM measurements with seated subjects and Kinect ROM measurements for standing subjects. The authors attributed this result to the limitation of the Kinect’s skeletal tracking, which is optimised for standing rather than sitting. Moreover, better accuracy for the Kinect has been reported for standing postures [136]. Therefore, adequate patient positioning and protocol standardisations are essential to reduce measurement error [105]. Suggested examples include placing coloured footprints on the floor and fixating the Kinect sensor bar [105].

Wearability and usability are two aspects to consider for implementing sensor-based human motion tracking devices in clinical practice. For the included studies, IMUs were most often positioned on the upper arm with additional placements on the sternum and wrist. Methods of fixation included double-sided adhesive tape with an elastic cohesive [100], an elastic belt [114] and, velcro straps [118]. Smartphone devices were attached by commercial armbands [110,111,116,120] or were hand-held by the examiners [95,113,122]. Most notably, no studies reported any calibration issues, and only one study [111] reported attachment difficulties.

### 4.3. Limitations

There were some limitations in this review. First, no additional search was performed for grey literature, and only studies written in English were included. Although authors identified an additional six reliability studies, they were excluded because they did not assess ICCs.

Second, the authors acknowledge the limitations of the revised COSMIN methodological quality tool, as it was primarily developed to assess risk of bias and not study design. Although more user-friendly than the original version, the omission of a sample size criterion leaves open a wider interpretation as to what constitutes an adequate sample size. Furthermore, no standards exist regarding the types of patients, examiners (well-trained or otherwise), and testing procedures. Future studies can apply other tools such as the modified GRADE (Grading of Recommendations Assessment, Development, and Evaluation) approach to address these issues [137]. Additionally, because the revised COSMIN guidelines are relatively new update, caution should be exercised when interpreting and comparing these results with prior studies that used the original COSMIN checklist.

Third, our meta-analysis was limited by the heterogeneity of the studies, given the variance in sample sizes, protocols, shoulder positions, and number of raters. Several studies did not report the 95% confidence intervals for ICCs. Furthermore, the calculation methods for ROM angles with the Kinect represents a potential source of difference across studies. Therefore, the general conclusions should be interpreted with caution.

Lastly, for reasons mentioned earlier, the authors did not examine validity, the degree to which a tool measures what it claims to measure. However, given the potential variety and the lack of any agreed-upon “gold standard” tool identified in the literature, a separate review is warranted to address validity. Reliability should always be interpreted with validity in mind to provide a complete assessment of the clinical appropriateness of a measuring tool.

#### Future Directions

Future reliability studies should focus on improving study design, with larger sample sizes (>80 participants) [138] and set recommended time intervals (2–7 days) between repeated measurements to increase confidence with results. Moreover, further investigations should report on absolute measures of reliability or measurement error to improve the overall risk of bias.

## 5. Conclusions

The primary result of our systematic review is that the Kinect and ambulatory sensor-based human motion tracking devices demonstrate moderate to good levels of intra- and inter-rater reliability to measure shoulder ROM. The assessment of reliability is an initial step in recommending a measuring tool for clinical use. Future research including the Kinect and other devices should investigate validity in well-designed, high-quality studies.

## Figures and Tables

**Table 1:** Characteristics of studies included in this review.

Author	Sample Size (*n*)	Age (yr) Mean (SD)	Males (%)	Inclusion Criteria	Rater (*n*, Profession)	Movement Assessed	Position	Device	Sessions (*n*)	Time Interval
Awan et al., 2002 [91]	56	Not reported	57.1	No history of neurologic disease, arthritis, connective tissue disorder, or shoulder/neck injury or surgery	2 PT MP	Passive • IR • ER	Supine	Digital inclinometer	2	90–120 min
Beshara et al., 2016 [92]	9	36.6 (±13.3)	33.3	No history of neurologic disease, arthritis, connective tissue disorder, or shoulder/neck injury or surgery	1 PT	Active • F • Abd	Standing	Microsoft Kinect (V.2) and Inertial sensors	2	7 days
Bonnechère et al., 2014 [93]	48	26 (±8)	62.5	Healthy adults	1	Active • Abd	Standing	Microsoft Kinect (V.1.5)	2	7 days
Cai et al., 2019 [94]	10	24.6 (±2.8)	100	No upper limb injuries or medication use that would have influenced their upper limb functions	1	Active • F • E • Abd • Add • IR • ER	Standing	Microsoft Kinect (V.2)	2	7 days
Chan et al., 2010 [95]	1	Not reported	100	Healthy, no pathology	2	Active • F • E • RER in 90° Abd	Standing Supine	iPod touch	2	Same day
Chen et al., 2020 [96]	10	Not reported	Not reported	Healthy, aged 20–70 yrs, no discomfort or limited ROM of shoulder in the last year	2 -1 PT -1 MP	Active • F • E • Abd • IR • ER	Standing	Inertial sensor (BoostFix)	1	Same day
Cools et al., 2014 [97]	30	22.1 (1.4)	50	No history of shoulder or neck pain or current participation in overhead sports on a competition level	2	Passive • ER • ER in 90° • Abd • IR in 90° • Abd • IR in forward • F	Sitting Supine	Digital inclinometer	2	10 s
Correll et al., 2018 [98]	42	32.3 (2.1)	71.4	Healthy, 18–75 yrs old, able to easily move between standing and supine positions, able to actively move at least one shoulder into 90° of glenohumeral abduction	2 PT student	Active • F • Abd • ER • IR	Supine	Digital inclinometer (HALO)	2	Same day
Çubukçu et al., 2020 [99]	40	22.1 (±3.1)	55	Healthy volunteers	1 PT	Active • F • E • Abd • ER • IR	Standing	Microsoft Kinect (V.2)	3	3 days
Cuesta-Vargas et al., 2016 [100]	37	56.1 (Healthy) 52.8 (Pathologic)	40.5	Healthy: no shoulder pain, negative NEER/Hawkin’s testPathologic: 18–75 yrs old, BMI 18–42	2 PT	Active • Abd	Standing	Inertial sensors (Inertia-Cube 3)- Sampling frequency 1000 Hz Smartphone (Nexus 4) 1280 × 768p resolution	3	2 days
Da Cunha Neto et al., 2018 [101]	10	Notreported	Not reported	Healthy	2	Active • F • E • Abd • Add	Standing	Microsoft Kinect (V.2)	2	Same day
De Baets et al., 2020 [102]	10	54 (±6)	57.1	Diagnosis of adhesive capsulitis in the past 6 months based on criterial described by the American Physical Therapy Association	2	Active • F • E • Abd • Add • IR • ER	Standing Seated	Inertial sensor (MCN Awinda motion capture system)-Sampling frequency 60 Hz	2	2–5 days
de Winter et al., 2004 [103]	155	47	35.5	Shoulder pain, 18–75 yrs, ability to co-operate (no dementia), sufficient knowledge of Dutch language	2 PT	Passive • Abd • ER	Seated Supine	Digital inclinometer (Cybex EDI 320)	1	1 h
Dougherty et al., 2015 [104]	90	23.5 (8.9)	40	18 yrs +, pain free shoulder movement, no history of shoulder pain in preceding 12 months	1 PT	Passive • FGH • F • Abd • GH • Abd • ER in neutral • Abd • ER in 90° • Abd • IR in 90° Abd	Seated Supine	Digital inclinometer	2	7 days
Hawi et al., 2014 [105]	7	Not Reported	Not Reported	Age 18+, free ROM without deficits	1	Active • FE • Abd • Add	Standing	Microsoft Kinect	2	Same day
Huber et al., 2015 [106]	10	22.1 (±0.9)	60	No shoulder pathology, pain-free	1	Active • F to 90° F to max • Abd to 90° • Sagittal F to 90° • Sagittal F to max • ER to max	Standing	Microsoft Kinect	1	Same day
Hwang et al., 2017 [107]	8	36.5 (±13.7)	Not Reported	Using a wheelchair for 1 yr, able to sit upright for at least 4 h/day, over 18 yrs old, use a wheelchair over 40 h/week	1	Active • F • E • Abd • Add	Seated	Microsoft Kinect (V.2)	2	Same day
Kolber et al., 2011 [108]	30	25.9 (3.1)	40	Asymptomatic adults	2 PT	Active • F • Abd • IR • ER	Seated Supine Prone	Digital inclinometer (Acumar)	2	2 days
Kolber et al., 2012 [109]	30	26 (4.2)	30	No cervical spine or upper extremity pain or recent shoulder surgery on dominant arm	2 PT student	Active • Scaption	Seated	Digital inclinometer (Acumar)	2	1 day
Lim et al., 2015 [110]	47	24.9 (±3.5)	59.6	No shoulder injuries or history of musculoskeletal and nervous system damage that could affect ROM, no pain around shoulder no performance of specialized shoulder muscle stretch or exercises or stretching in preceding 6 months	2 PT	Passive • Abd	Supine Side-lying	Smartphone (iPhone 5)	2	2 days
Mejia-Hernandez et al., 2018 [111]	75	46	72	Older than 18 yrs, documented current shoulder diseases	2 MP	Active & Passive • Forward F • Abd • GH • Abd • IR • ER	Seated Supine	Smartphone (iPhone 5)	2	Same day
Milgrom et al., 2016 [112]	5	Not reported	80	Possess ability to self-propel a manual wheelchair, uses a wheelchair for at least 75% of daily activities, ≥18 yrs of age	3 Kinect sensors “individual rater”	Active • F • Abd	Seated	Microsoft Kinect (V.1.8)	2	Same day
Mitchell et al., 2014 [113]	94	Not reported	0	No shoulder pathology	5 -2 PT -3 PT students	Active • ER	Supine	Smartphone (iPhone 4)	2	At least 15 min (<30 min)
Picerno et al., 2015 [114]	45	M: 27 (±8) F: 22 (±3)	55.6	No previous or current shoulder impairment, no involvement in competitive sports at a professional level	1	Active • Abd	Seated	Inertial sensor (FreeSense)-Sampling frequency 200 Hz	2	Same day
Poser et al., 2015 [115]	23	44	39.1	Asymptomatic people who are attending a Pilates gym	3 PT	Active • ER • IR • Abd • Hor • Add	Supine Seated Side-lying	Digital Inclinometer (J-Tech)	2	Days (unspecific)
Ramos et al., 2019 [116]	54	26.3 (6) Healthy 25 (6) Shoulder pain	25.9	Healthy: Not reported Shoulder pain: Symptoms for at least 6 months and positive clinical tests for shoulder impingement	1	Active • F • Scaption	Seated	Mobile application (iPod)	2	7 days
Rigoni et al., 2019 [117]	30	32.8	40	Healthy volunteers	2	Active • F • Abd • ER • IR	Standing	Inertial Sensor (Biokin)	1	Same day
Schiefer et al., 2015 [118]	20	37.4 (±9.9)	70	Healthy subjects without or with known functional deficits, free of musculoskeletal complaints for at least one week before examination	3 MP	Passive • ER • IR	Not reported	Inertial Sensor (CUELA system)	1	1 day
Scibek et al., 2013 [119]	11	21.4 (±1.4)	55.6	Healthy, reporting no history of neck, upper extremity pathology in the last six months	Not reported	Active • F • GH • F • Abd	Seated	Digital inclinometer (Pro 360, Baseline)	2	12–48 h
Shin et al., 2012 [120]	41	52.7 (±17.5)	48.8	Unilateral symptomatic shoulders	3 MP	Active & Passive • Forward F • Abd • ER • ER at 90° • Abd • IR at 90° • Abd	StandingSupine	Smartphone (Galaxy S)	2	Same day
Walker et al., 2016 [121]	17	17 (±3)	47	Healthy, competitive swimmers, at least five swim sessions per week	2 PT	Active • EL • EI • RER • Abd in IR	SupineStanding	Digital inclinometer (Dualer, J-Tech)	2	30 min
Werner et al., 2014 [122]	24	Not reported	37.5	Healthy and symptomatic shoulders, college students	5 -4 MP -1 Medical student	Active • Forward F • Abd • ER at 0° • ER at 90° • IR at 90° • Abd	SupineStanding	Smartphone (iPhone)	2	Same day

Abd = abduction, Add = adduction, ELE = elevation, ER = external rotation, E = extension, ER= external rotation, F = flexion, GH = Glenohumeral, Hor = horizontal, IR = internal rotation, Max = maximum, MP = medical physician, PT = physiotherapist, ROM = range of motion.

**Table 2:** Intra-rater and Inter-rater reliability (95% CI) for measurement of shoulder range of motion by device and movement direction.

Device	Author	Intra-Rater Reliability	Inter-Rater Reliability	Level of Reliability
Microsoft Kinect				
Shoulder				
Flexion	Da Cuncha Neto et al. (2018) Hawi et al. (2014) Huber et al. (2015) Hwang et al. (2017) Milgrom et al. (2016) Çubukçu et al. (2020) Cai et al. (2019)	ICC 0.97 ICC 0.99 ICC 0.37, 0.85, 0.84, 0.95 ICC 0.96 (0.83–0.98), 0.92 (0.89–0.95) ICC 0.85 ICC 0.93, 0.99, 0.97. 0.96	ICC 0.91 ICC 0.97 (0.84–1.00)	*Good* *Good* *Poor–Good* *Good* *Good* *Moderate* *Good*
Extension	Da Cuncha Neto et al. (2018) Hawi et al. (2014) Hwang et al. (2017) Çubukçu et al. (2020) Cai et al. (2019)	ICC 0.97 ICC 0.99 ICC 0.96 (0.83–0.98), 0.92 (0.89–0.95) ICC 0.62 ICC 0.93, 0.99, 0.97, 0.96	ICC 0.97	*Good* *Good* *Good* *Poor* *Good*
Abduction	Bonnechère et al. (2014) Hawi et al. (2014) Huber et al. (2015) Hwang et al. (2017) Milgrom et al. (2016) Cai et al. (2019)	ICC 0.73 ICC 0.96 ICC 0.76 ICC 0.92 (0.89–0.93), 0.96 (0.86–0.96) ICC 0.70, 0.75, 0.84, 0.82	ICC 0.94 (0.72–0.99)	*Moderate* *Good* *Moderate* *Good* *Good* *Moderate*
Adduction	Hawi et al. (2014) Hwang et al. (2017) Cai et al. (2019)	ICC 0.99 ICC 0.92 (0.89–0.93), 0.96 (0.86–0.96) ICC 0.70, 0.75, 0.84, 0.82		*Good* *Good* *Moderate*
External rotation	Huber et al. (2015) Çubukçu et al. (2020) Cai et al. (2019)	ICC 0.98 ICC 0.87 ICC 0.93, 0.75, 0.90, 0.60		*Good* *Good* *Moderate–Good*
Internal rotation	Çubukçu et al. (2020) Cai et al. (2019)	ICC 0.97 ICC 0.93, 0.75, 0.90, 0.60		*Good* *Moderate–Good*
Microsoft Kinect & Inertial Sensor				
Shoulder				
Flexion	Beshara et al. (2016)	ICC 0.84 (0.45–0.96), 0.93 (0.72–0.98)		*Moderate–Good*
Abduction	Beshara et al. (2016)	ICC 0.52 (-0.17–0.87, 0.85 (0.47–0.96)		*Poor–Moderate*
Inertial Sensor				
Shoulder				
Flexion	Rigoni et al. (2019) Chen et al. (2020) De Baets et al. (2020)	ICC 0.68, 0.87, 0.91	ICC 0.88 (0.80–0.92) ICC 0.90 (0.83–0.94), 0.95 (0.92–0.97) ICC 0.74, 0.83, 0.84	*Good* *Good* *Moderate–Good (Intra-rater)* *Moderate (Inter-rater)*
Extension	Chen et al. (2020) De Baets et al. (2020)	ICC 0.68, 0.87, 0.91	ICC 0.77 (0.64–0.87), 0.80 (0.68–0.89) ICC 0.74, 0.83, 0.84	*Moderate* *Moderate–Good (Intra-rater)* *Moderate (Inter-rater)*
Abduction	Cuesta-Vargas et al. (2016) Picerno et al. (2015) Rigoni et al. (2019) Chen et al. (2020) De Baets et al. (2020)	ICC 0.78 (0.40–0.93), 0.98 (0.94–0.99) 0.99 (0.98–0.99), 0.96 (0.93–0.98) ICC 0.96 (0.93–0.98) ICC 0.73, 0.95	ICC 0.49 (0.08–0.82), 0.99 (0.98–1.00), 0.99 (0.99–1.00) ICC 0.88 (0.81–0.93) ICC 0.97 (0.95–0.98), 0.98 (0.96–0.99) ICC 0.74, 0.80, 0.93	*Moderate–Good (Intra-rater)* *Poor–Good (Inter-rater)* *Good* *Good* *Good* *Moderate–Good*
Adduction	De Baets et al. (2020)	ICC 0.73, 0.95	ICC 0.74, 0.80, 0.93	*Moderate–Good*
External rotation	Schiefer et al. (2015) Rigoni et al. (2019) Chen et al. (2020) De Baets et al. (2020)	ICC 0.85, 0.87, 0.89, 0.90	ICC 0.71, 0.76, 0.81, 0.86 ICC 0.84 (0.74–0.90) ICC 0.95 (0.92–0.97), 0.96 (0.93–0.98) ICC 0.38, 0.84, 0.73, 0.87	*Moderate–Good* *Good* *Good* *Moderate–Good (Intra-rater)* *Poor–Good (Inter-rater)*
Internal rotation	Schiefer et al. (2015) Rigoni et al. (2019) Chen et al. (2020) De Baets et al. (2020)	ICC 0.85, 0.87, 0.89, 0.90	ICC 0.68, 0.78, 0.87, 0.98 ICC 0.71 (0.56–0.82) ICC 0.91 (0.86–0.95), 0.97 (0.94–0.98) ICC 0.38, 0.84, 0.73, 0.87	*Moderate–Good* *Moderate* *Good* *Moderate–Good (Intra-rater)* *Poor–Good (Inter-rater)*
Smartphone/Mobile App				
Shoulder				
Flexion	Chan et al. (2010) Shin et al. (2012) Werner et al. (2014) Mejia-Hernandez et al. (2018) Ramos et al. (2019)	ICC 0.99 ICC 0.97 (0.95–0.99), 0.96 (0.92–0.98) 0.99 (0.98–0.99), 0.99 (0.99–1.00) ICC −0.21, −0.19, 0.01, 0.16, 0.27, 0.40 0.47, 0.50, 0.53, 0.56, 0.60, 0.71, 0.76, 0.82	ICC 0.99 ICC 0.73 (0.59–0.83), 0.74 (0.61–0.84), 0.83 (0.73–0.90), 0.84 (0.74–0.90) ICC 0.75 (0.61–0.84), 0.97 (0.90–0.99) ICC 0.99 (0.98–0.99) ICC 0.06, 0.18, 0.19, 0.22, 0.25, 0.27, 0.30, 0.36, 0.40, 0.44, 0.47, 0.49, 0.68, 0.69	*Good* *Good (intra-rater)* *Moderate–Good (inter-rater)* *Moderate–Good* *Good* *Poor–Moderate*
Abduction	Lim et al. (2015) Shin et al. (2012) Werner et al. (2014) Mejia-Hernandez et al. (2018)	ICC 0.72, 0.89, 0.95, 0.97 ICC 0.96, 0.97, 0.99	ICC 0.79, 0.94 ICC 0.70, 0.72, 0.78, 0.79 ICC 0.72 (0.58–0.83), 0.91 (0.68–0.97) ICC 0.99 (0.99–1.00)	*Moderate–Good* *Good (intra-rater)* *Moderate (inter-rater)* *Moderate–Good* *Good*
Glenohumeral abduction	Mejia-Hernandez et al. (2018)		ICC 0.98 (0.97–0.99), 0.97 (0.95–0.99)	*Good*
External rotation	Chan et al. (2010) Mitchell et al. (2014) Shin et al. (2012) Werner et al. (2014) Mejia-Hernandez et al. (2018)	ICC 0.94, 0.96 ICC 0.79 (0.70–0.86) ICC 0.95, 0.97, 0.98	ICC 0.88, 0.98 ICC 0.94 (0.87–0.98) ICC 0.76, 0.77, 0.78, 0.89, 0.90 ICC 0.85 (0.57–0.95), 0.86 (0.79–0.92), 0.88 (0.66–0.96) ICC 0.99 (0.98–0.99)	*Good* *Moderate (intra-rater)* *Good (inter-rater)* *Good (intra-rater)* *Moderate–Good (inter-rater)* *Good* *Good*
Internal rotation	Shin et al. (2012) Werner et al. (2014) Mejia-Hernandez et al. (2018)	ICC 0.79, 0.97, 0.90, 0.93 0.99	ICC 0.63, 0.66, 0.67, 0.68 ICC 0.81 (0.70–0.88), 0.86 (0.48–0.93) ICC 0.98 (0.97–0.99), 0.98 (0.96–0.98)	*Moderate–Good* *Good* *Good*
Scaption	Ramos et al. (2019)	ICC −0.04, 0.10, 0.12, 0.31, 0.32, 0.39, 0.40, 0.45, 0.47, 0.52, 0.57, 0.63	ICC −0.17, −0.06, 0.03, 0.07, 0.23, 0.26, 0.27, 0.28, 0.36, 0.45, 0.54, 0.73, 0.75, 0.81	*Poor–Moderate (intra-rater)* *Poor–Good (inter-rater)*
Digital Inclinometer/Goniometer				
Shoulder				
Flexion	Dougherty et al. (2015) Kolber et al. (2011) Scibek et al. (2013) Correll et al. (2018)	ICC 0.77, 0.82 ICC 0.83 ICC 0.67, 0.80, 0.90, 0.92, 0.96 ICC 0.86, 0.88	ICC 0.58 ICC 0.18, 0.33, 0.50, 0.62, 0.68, 0.72, 0.76, 0.78, 0.85 ICC 0.89	*Moderate* *Moderate (intra-rater)* *Poor (inter-rater)* *Moderate–Good (intra-rater)* *Poor–Moderate (inter-rater)* *Good*
Elevation	Walker et al. (2016)	ICC 0.91, 0.92, 0.93, 0.95		*Good*
Glenohumeral flexion	Dougherty et al. (2015) Scibek et al. (2013)	ICC 0.75, 0.77 ICC 0.75, 0.92, 0.94, 0.96, 0.99	ICC 0.14, 0.35, 0.43, 0.63, 0.65, 0.69, 0.72, 0.83	*Moderate* *Moderate–Good (intra-rater)* *Poor–Good (inter-rater)*
Abduction	deWinter et al. (2004) Kolber et al. (2011) Poser et al. (2015) Dougherty et al. (2015) Scibek et al. (2013) Walker et al. (2016) Correll et al. (2018)	ICC 0.91 ICC 0.83, 0.92, 0.93, 0.96 ICC 0.73, 0.76 ICC 0.91, 0.94, 0.95, 0.96, 0.97, 0.99 ICC 0.89, 0.90, 0.91, 0.94 ICC 0.86, 0.91	ICC 0.28, 0.78, 0.83 ICC 0.95 ICC 0.27, 0.32, 0.40, 0.60, 0.63, 0.64 ICC 0.48, 0.56, 0.58, 0.62, 0.65, 0.68, 0.70, 0.80, 0.83 ICC 0.93	*Poor–Good* *Moderate* *Moderate–Good (intra-rater)* *Poor–Moderate (inter-rater)* *Moderate* *Good (intra-rater)* *Poor–Good (inter-rater)* *Good* *Good*
Glenohumeral abduction	Dougherty et al. (2015)	ICC 0.60, 0.75		*Moderate*
Horizontal abduction	Poser et al. (2015)	ICC 0.66, 0.81, 0.91, 0.94, 0.96	ICC 0.17, 0.18, 0.24, 0.28, 0.31	*Moderate–Good (intra-rater)* *Poor (inter-rater)*
Digital Inclinometer/Goniometer External rotation	Awan et al. (2002) Cools et al. (2014) deWinter et al. (2004) Kolber et al. (2011) Poser et al. (2015) Dougherty et al. (2015) Walker et al. (2016) Correll et al. (2018)	ICC 0.58, 0.67 ICC 0.98, 0.95, 0.98 ICC 0.94 ICC 0.93, 0.94, 0.96, 0.97 ICC 0.28, 0.61, 0.66, 0.64, 0.68, 0.71 ICC 0.90, 0.94, 0.95 ICC 0.89, 0.90	ICC 0.41, 0.51 ICC 0.98 ICC 0.56, 0.88, 0.90 ICC 0.88 ICC 0.70, 0.71, 0.72, 0.73, 0.76, 0.77 ICC 0.98	*Poor–Moderate (intra-rater)* *Poor (inter-rater)* *Good* *Poor–Good* *Good* *Good (intra-rater)* *Moderate (inter-rater)* *Poor–Moderate* *Good* *Good*
Internal rotation	Awan et al. (2002) Cools et al. (2014) Kolber et al. (2011) Poser et al. (2015) Dougherty et al. (2015) Walker et al. (2016) Correll et al. (2018)	ICC 0.64, 0.65, 0.72 ICC 0.89, 0.98, 0.99 ICC 0.87 ICC 0.91, 0.92, 0.94, 0.96, 0.97 ICC 0.64, 0.68 ICC 0.85, 0.90, 0.93, 0.96 ICC 0.82, 0.85	ICC 0.50, 0.52, 0.62, 0.66 ICC 0.96, 0.98 ICC 0.93 ICC 0.63, 0.66, 0.73, 0.76, 0.78 ICC 0.96	*Poor–Moderate* *Good* *Good* *Good (intra-rater)* *Moderate (inter-rater) Poor–Moderate* *Moderate–Good* *Moderate–Good*
Scaption	Kolber et al. (2012)	ICC 0.88	ICC 0.89	*Good*

ICC = intra-class correlation coefficient. Level of reliability determined by the criteria identified by Swinkels et al. [87].

**Table 3:** Assessment of reliability using the COSMIN standards for studies on reliability checklist.

**Items**	**First Author and Year**										
	**Awan (2002)**	**Beshara (2016)**	**Bonnechère (2014)**	**Cai (2019)**	**Chan (2010)**	**Chen (2020)**	**Cools (2014)**	**Correll (2018)**	**Çubukçu (2020)**	**Cuesta-Vargas (2016)**	**Da Cunha Neto (2018)**
1. Were patients stable in the time between repeated measurements on the construct to be measured?	VG	VG	VG	VG	VG	VG	VG	VG	VG	VG	A
2. Was the time interval between the repeated measurements appropriate?	A	VG	VG	VG	A	A	A	A	VG	VG	A
3. Were the measurement conditions similar for the repeated measurements–except for the condition being evaluated as a source of variation?	VG	VG	VG	VG	A	VG	VG	VG	VG	VG	A
4. Did the professional(s) administer the measurement without knowledge of scores or values of other repeated measurement(s) in the same patients?	VG	VG	VG	A	VG	VG	VG	VG	A	A	A
5. Did the professionals(s) assign scores or determine values without knowledge of scores or values of other repeated measurements(s) in the same patients?	VG	VG	VG	A	VG	VG	VG	VG	A	A	A
6. Were there any other important flaws in the design or statistical methods of the study?	D	D	VG	A	I	A	VG	VG	VG	VG	A
7. For continuous scores: was an intraclass correlation (ICC) calculated?	A	VG	A	VG	A	VG	VG	VG	VG	VG	A
8. For ordinal scores: was a (weighted) kappa calculated?	N/A	N/A	N/A	N/A	N/A	N/A	N/A	N/A	N/A	N/A	N/A
9. For dichotomous/nominal scores: was Kappa calculated for each category against the other categories combined?	N/A	N/A	N/A	N/A	N/A	N/A	N/A	N/A	N/A	N/A	N/A
**Overall Score**	D	D	A	A	I	A	A	A	A	A	A
**Items**	**First Author and Year**										
	**De Baets (2020)**	**deWinter (2004)**	**Dougherty (2015)**	**Hawi (2014)**	**Huber (2015)**	**Hwang (2017)**	**Kolber (2011)**	**Kolber (2012)**	**Lim (2015)**	**Mejia-Hernandez (2018)**	**Milgrom (2016)**
1 Were patients stable in the time between repeated measurements on the construct to be measured?	VG	VG	A	VG	VG	VG	VG	VG	VG	VG	VG
2. Was the time interval between the repeated measurements appropriate?	A	A	VG	A	A	A	A	VG	VG	A	A
3. Were the measurement conditions similar for the repeated measurements–except for the condition being evaluated as a source of variation?	VG	VG	VG	VG	VG	VG	VG	VG	VG	VG	VG
4. Did the professional(s) administer the measurement without knowledge of scores or values of other repeated measurement(s) in the same patients?	A	A	VG	A	VG	A	VG	VG	VG	VG	A
5. Did the professionals(s) assign scores or determine values without knowledge of scores or values of other repeated measurements(s) in the same patients?	A	A	VG	A	VG	A	VG	VG	VG	VG	A
6. Were there any other important flaws in the design or statistical methods of the study?	A	VG	VG	D	A	D	VG	VG	VG	VG	D
7. For continuous scores: was an intraclass correlation (ICC) calculated?	VG	A	A	VG	VG	A	VG	VG	VG	A	A
8. For ordinal scores: was a (weighted) kappa calculated?	N/A	N/A	N/A	N/A	N/A	N/A	N/A	N/A	N/A	N/A	N/A
9. For dichotomous/nominal scores: was Kappa calculated for each category against the other categories combined?	N/A	N/A	N/A	N/A	N/A	N/A	N/A	N/A	N/A	N/A	N/A
**Overall Score**	A	A	A	D	A	D	A	VG	VG	A	D
**Items**	**First Author and Year**										
	**Mitchell (2014)**	**Picerno (2015)**	**Poser (2015)**	**Ramos (2019)**	**Rigoni (2019)**	**Schiefer (2015)**	**Scibek (2013)**	**Shin (2012)**	**Walker (2016)**	**Werner (2014)**	
1. Were patients stable in the time between repeated measurements on the construct to be measured?	VG	VG	VG	VG	VG	VG	VG	VG	VG	VG	
2. Was the time interval between the repeated measurements appropriate?	A	A	VG	VG	A	A	A	A	A	A	
3. Were the measurement conditions similar for the repeated measurements–except for the condition being evaluated as a source of variation?	VG	VG	VG	VG	VG	VG	VG	VG	VG	VG	
4. Did the professional(s) administer the measurement without knowledge of scores or values of other repeated measurement(s) in the same patients?	VG	A	A	A	VG	VG	A	VG	VG	VG	
5. Did the professionals(s) assign scores or determine values without knowledge of scores or values of other repeated measurements(s) in the same patients?	VG	A	A	A	VG	VG	A	VG	VG	VG	
6. Were there any other important flaws in the design or statistical methods of the study?	VG	VG	A	VG	VG	A	A	VG	A	A	
7. For continuous scores: was an intraclass correlation (ICC) calculated?	VG	VG	VG	A	VG	VG	VG	VG	VG	VG	
8. For ordinal scores: was a (weighted) kappa calculated?	N/A	N/A	N/A	N/A	N/A	N/A	N/A	N/A	N/A	N/A	
9. For dichotomous/nominal scores: was Kappa calculated for each category against the other categories combined?	N/A	N/A	N/A	N/A	N/A	N/A	N/A	N/A	N/A	N/A	
**Overall Score**	A	A	A	A	A	A	A	A	A	A	

Abbreviations: VG: very good; A: adequate, D: doubtful; I: inadequate; N/A: not applicable.

**Table 4:** Assessment of measurement error using the COSMIN standards for studies on measurement error checklist.

**Items**	**First Author and Year**										
	**Awan (2002)**	**Beshara (2016)**	**Bonnechère (2014)**	**Cai (2019)**	**Chan (2010)**	**Chen (2020)**	**Cools (2014)**	**Correll (2018)**	**Çubukçu (2020)**	**Cuesta-Vargas (2016)**	**Da Cunha Neto (2018)**
1 Were patients stable in the time between repeated measurements on the construct to be measured?	VG	VG	VG	VG	VG	VG	VG	VG	VG	VG	A
2. Was the time interval between the repeated measurements appropriate?	A	VG	A	VG	A	A	A	A	VG	VG	D
3. Were the measurement conditions similar for the repeated measurements–except for the condition being evaluated as a source of variation?	VG	VG	VG	VG	A	VG	VG	VG	VG	VG	A
4. Did the professional(s) administer the measurement without knowledge of scores or values of other repeated measurement(s) in the same patients?	VG	VG	VG	A	VG	VG	VG	VG	A	A	A
5. Did the professionals(s) assign scores or determine values without knowledge of scores or values of other repeated measurements(s) in the same patients?	VG	VG	VG	A	VG	VG	VG	VG	A	A	A
6 Were there any other important flaws in the design or statistical methods of the study?	D	D	VG	D	I	VG	VG	VG	VG	VG	VG
7. For continuous scores: was the Standard Error of Measurement (SEM), Smallest Detectable Change (SDC), Limits of Agreement (LoA) or Coefficient of Variation (CV) calculated?	I	VG	VG	I	I	VG	VG	VG	VG	I	I
8. For dichotomous/nominal/ordinal scores: was the percentage specific (e.g., positive and negative) agreement calculated?	N/A	N/A	N/A	N/A	N/A	N/A	N/A	N/A	N/A	N/A	N/A
**Overall Score**	I	D	A	I	I	A	A	A	A	I	I
**Items**	**First Author and Year**										
	**De Baets (2020)**	**deWinter (2004)**	**Dougherty (2015)**	**Hawi (2014)**	**Huber (2015)**	**Hwang (2017)**	**Kolber (2011)**	**Kolber (2012)**	**Lim (2015)**	**Mejia-Hernandez (2018)**	**Milgrom (2016)**
1. Were patients stable in the time between repeated measurements on the construct to be measured?	VG	VG	A	VG	VG	VG	VG	VG	VG	VG	VG
2. Was the time interval between the repeated measurements appropriate?	A	A	VG	A	A	A	A	VG	VG	A	A
3. Were the measurement conditions similar for the repeated measurements–except for the condition being evaluated as a source of variation?	VG	VG	VG	A	VG	VG	VG	VG	VG	VG	VG
4. Did the professional(s) administer the measurement without knowledge of scores or values of other repeated measurement(s) in the same patients?	A	A	VG	A	VG	A	VG	VG	VG	VG	A
5. Did the professionals(s) assign scores or determine values without knowledge of scores or values of other repeated measurements(s) in the same patients?	A	A	VG	A	VG	A	VG	VG	VG	VG	A
6. Were there any other important flaws in the design or statistical methods of the study?	VG	VG	VG	D	VG	D	VG	VG	D	VG	D
7. For continuous scores: was the Standard Error of Measurement (SEM), Smallest Detectable Change (SDC), Limits of Agreement (LoA) or Coefficient of Variation (CV) calculated?	VG	N/A	VG	I	VG	VG	VG	VG	I	VG	I
8. For dichotomous/nominal/ordinal scores: was the percentage specific (e.g., positive and negative) agreement calculated?	N/A	VG	A	N/A	N/A	N/A	N/A	N/A	N/A	N/A	N/A
**Overall Score**	A	A	A	I	A	D	A	VG	I	A	I
**Items**	**First Author and Year**										
	**Mitchell (2014)**	**Picerno (2015)**	**Poser (2015)**	**Ramos (2019)**	**Rigoni (2019)**	**Schiefer (2015)**	**Scibek (2013)**	**Shin (2012)**	**Walker (2016)**		**Werner (2014)**
1. Were patients stable in the time between repeated measurements on the construct to be measured?	VG	VG	VG	VG	VG	VG	VG	VG	VG		VG
2. Was the time interval between the repeated measurements appropriate?	A	A	VG	VG	A	A	A	A	A		A
3. Were the measurement conditions similar for the repeated measurements–except for the condition being evaluated as a source of variation?	VG	VG	VG	VG	VG	VG	VG	VG	VG		VG
4. Did the professional(s) administer the measurement without knowledge of scores or values of other repeated measurement(s) in the same patients?	VG	A	A	A	VG	VG	A	VG	VG		VG
5. Did the professionals(s) assign scores or determine values without knowledge of scores or values of other repeated measurements(s) in the same patients?	VG	A	A	A	VG	VG	A	VG	VG		VG
6. Were there any other important flaws in the design or statistical methods of the study?	VG	VG	VG	VG	VG	VG	D	VG	VG		VG
7. For continuous scores: was the Standard Error of Measurement (SEM), Smallest Detectable Change (SDC), Limits of Agreement (LoA) or Coefficient of Variation (CV) calculated?	I	I	VG	VG	VG	VG	I	VG	VG		VG
8. For dichotomous/nominal/ordinal scores: was the percentage specific (e.g., positive and negative) agreement calculated?	N/A	N/A	N/A	N/A	N/A	N/A	N/A	N/A	N/A		N/A
**Overall Score**	I	I	A	A	A	A	I	A	A		A

Abbreviations: VG: very good; A: adequate, D: doubtful; I = inadequate; N/A: not applicable.

**Table 5:** Anatomical landmarks by device.

Device	Author	Anatomical Landmarks
Microsoft Kinect	Bonnechère et al. (2014)	Shoulder girdle centre, elbow, wrist, hand
	Hawi et al. (2014)	Shoulder centre and elbow
	Huber et al. (2015)	Positions of shoulder and elbow joints relative to the trunk for flexion and abduction. Position of elbow and hand relative to trunk for external rotation
	Milgrom et al. (2016)	Angle between the humerus vector (shoulder to elbow) and the torso vector (neck to shoulder midpoint)
	Cai et al. (2019)	X = Unit vector perpendicular to the Y-axis and the Z-axis pointing anteriorly, Y: Unit vector going from the elbow joint center to the shoulder joint center, Z: Unit vector perpendicular to the plane formed by the Y-axis of the upper arm and the long axis vector of the forearm.
Microsoft Kinect & Inertial Sensor	Beshara et al. (2016)	2 3D vectors, a vector from shoulder joint centre (below the acromion process) to the elbow centre (between the medical and lateral epicondyles). A vector from shoulder joint centre defined as a point on the 6th rib along the midaxillary line of the trunk.
Inertial Sensor	Cuesta-Vargas et al. (2016)	Middle third of the humerus slightly posterior and in the flat part of the sternum
	Picerno et al. (2015)	Arbitrary point of the upper arm
	Schiefer et al. (2015)	Laterally on the upper arms and on the forearms close to the wrist, on the dorsum of the hand. Sensors were placed in the middle of the segments.
	Rigoni et al. (2019)	10 cm distal to the lateral epicondyle
	De Baets et al. (2020)	*Sternal sensor:* positioned on flat central part of the sternum, the *scapular sensor* halfway between the trigonum and the acromial angle, in alignment with the upper edge of the scapular spine. *Humeral sensor:* at the central third of the humerus, slightly posterior, at the level of the deltoid insertion. *Lower arm sensor:* positioned on the dorsal side, just proximal of the line between the radial and ulnar styloid process.
Smartphone/mobile app	Chan et al. (2010)	Acromion, humeral axis.
	Lim et al. (2015)	Front centre of humerus.
	Mitchell et al. (2014)	Superior border of the mid-ulna.
	Ramos et al. (2019)	Attached below the deltoid muscle origin
	Mejia-Hernandez et al. (2018)	Distal portion of the humerus for seated movements. Wrist for supine movements.
	Shin et al. (2012)	Ventral side of the patient’s forearm at the wrist level.
Digital Inclinometer	Dougherty et al. (2015)	*Shoulder flexion:* the anterior aspect of the arm, aligned parallel to the humerus. *Shoulder abduction:* The lateral aspect of the arm, aligned parallel to the humerus. *External Rotation:* The anterior aspect of the participant’s forearm. *Internal Rotation:* The posterior aspect of the participant’s forearm.
	Kolber et al. (2011)	*Flexion:* Distal arm proximal to the elbow. *Abduction:* Distal arm proximal to the elbow. *External rotation:* Distal forearm just proximal to the wrist. *Internal rotation:* Distal forearm just proximal to the wrist.
	Kolber et al. (2012)	*Scaption:* Superior portion of the humeral shaft proximal to the elbow.
	Poser et al. (2015)	*Abduction:* lateral and distal face of the humerus, with the inferior edge set at the beginning of the medial epicondyle. *Horizontal adduction:* spine of scapular and posterior face of the humerus, touching the olecranon.
	Scibek et al. (2013)	*Flexion and Abduction:* Shaft of the humerus
	Walker et al. (2016)	*Shoulder internal/external rotation:* 5 cm distal to the olecranon process of the elbow. *Combined elevation:* Just below the deltoid insertion with the face of the inclinometer in the coronal plane of movement. *Shoulder abduction in internal rotation:* Just below the deltoid insertion with the face of the inclinometer in the coronal plane of movement.
	Correll (2018)	*Shoulder flexion:* the lateral aspect of the greater tubercle, the midaxillary line of the thorax and the lateral midline of the humerus, lateral epicondyle of the humerus or the olecranon process. *Abduction:* anterior aspect of the acromial process, midline of the anterior aspect of the sternum and the anterior midline of the humerus. *External/Internal rotation:* the olecranon process, the ulna and ulnar styloid.

## Data Availability

The datasets presented in this study are openly available in [96,97,102,106].

## Supplementary Materials

The following are available online at https://www.mdpi.com/article/10.3390/s21248186/s1. Supplementary S1: Full search strategy.
