# Supplementary material for: The Reliability of the Microsoft Kinect and Ambulatory Sensor-Based Motion Tracking Devices to Measure Shoulder Range-of-Motion: A Systematic Review and Meta-Analysis
Source: Sensors (Basel). 2021 Dec 8;21(24):8186. doi: 10.3390/s21248186 (PMC8705315; doi:10.3390/s21248186)
Supplement: Supplementary file 1 [file sensors-21-08186-s001.zip › sensors-1476192-supplementary.pdf]

eAddenda

Beshara P., Anderson D.B, Pelletier M. and Walsh W.R.

The reliability of the Microsoft Kinect and ambulatory sensor-based motion tracking devices to measure shoulder range-of-motion: A systematic review

Supplementary S1: Full search strategy

Databases: Medline, Embase, EmCare, CINAHL, SportDiscus, Compendex, IEEE Xplore, Web of Science, Proquest Science & Technology, Scopus, Pubmed

Using the search terms below, the full holdings of Medline, Embase, CINAHL SportDiscus, Compendex, Ieee xplore, Web of Science, Proquest Science & Technology Scopus and Pubmed were searched from their earliest records to December 2020. The search strategy for each database is described below. All results were exported to endnote and duplicates were removed.

Medline search strategy

1. microsoft kinect.mp.
2. kinect.mp.
3. "Range of Motion, Articular"/
4. software/ or mobile applications/
5. 3 and 4
6. MONITORING, AMBULATORY/
7. (motion tracking adj6 (technology or device* or software or sensor*)).mp. [mp=title, abstract, original title, name of substance word, subject heading word, floating sub-heading word, keyword heading word, protocol supplementary concept word, rare disease supplementary concept word, unique identifier, synonyms]
8. digital inclinometer.mp.
9. inertial sensor*.mp.
10. SMARTPHONE/
11. iphone.mp.
12. Wearable Electronic Devices/
13. Accelerometry/
14. Magnetometry/ or magnetometer.mp.
15. gyroscope.mp.
16. SHOULDER/ or SHOULDER JOINT
17. 1 OR 2 OR 5 OR 6 OR 7 OR 8 OR 9 OR 10 OR 11 OR 12 OR 13 OR 14 OR 15
18. 16 AND 17

Embase search strategy

1. microsoft kinect.mp. or motion analysis system/
2. kinect.mp.
3. "range of motion"/
4. software/

5. 3 and 4
6. Ambulatory monitoring/
7. (motion tracking adj6 (technology or device* or software or sensor*)).mp. [mp=title, abstract, original title, name of substance word, subject heading word, floating sub-heading word, keyword heading word, protocol supplementary concept word, rare disease supplementary concept word, unique identifier, synonyms]
8. digital inclinometer.mp.
9. smartphone/
10. iphone.mp.
11. wearable electronic devices.mp.
12. inertial sensor*.mp.
13. accelerometer/ or accelerometry /
14. magnetometer.mp. or magnetometer/
15. gyroscope.mp.
16. shoulder/
17. 1 OR 2 OR 5 OR 6 OR 7 OR 8 OR 9 OR 10 OR 11 OR 12 OR 13 OR 14 OR 15
18. 16 AND 17

Emcare search strategy

1. microsoft kinect.mp.
2. kinect.mp.
3. "range of motion"/
4. software.mp.
5. 3 and 4
6. ambulatory monitoring/
7. (motion tracking adj6 (technology or device* or software or sensor*)).mp. [mp=title, abstract, original title, name of substance word, subject heading word, floating sub-heading word, keyword heading word, protocol supplementary concept word, rare disease supplementary concept word, unique identifier, synonyms]
8. digital inclinometer.mp.
9. inertial sensor*.mp.
10. smartphone/
11. iphone.mp.
12. wearable electronic devices.mp.
13. accelerometer/ or accelerometry /
14. magnetometer/
15. gyroscope.mp.
16. shoulder/
17. 1 OR 2 OR 5 OR 6 OR 7 OR 8 OR 9 OR 10 OR 11 OR 12 OR 13 OR 14 OR 15
18. 16 AND 17

CINAHL and SportDiscus search strategy

1. Search field for "kinect" OR "microsoft kinect" OR ("motion tracking" N6 technology) OR digital inclinometer OR inertial sensor OR smartphone OR iphone OR accelerometer OR magnetometer OR gyroscope
2. Search field for “shoulder”
3. Search field for “range of motion”
4. 1 AND 2 AND 3

Compendex search strategy

1. Search all fields for Kinect OR microsoft Kinect OR motion tracking N6 technology OR digital inclinometer* OR inertial sensor* OR smartphone OR iphone OR accelerometer OR magnetometer OR magnetometer* OR gyroscope*
2. Search all fields for “shoulder”
3. Search all fields for “range of motion”
4. 1 AND 2 AND 3

IEEE Xplore search strategy

1. Search field topic for kinect OR microsoft kinect OR motion tracking N6 technology OR digital inclinometer* OR inertial sensor* OR smartphone OR iphone OR accelerometer OR magnetometer OR magnetometer* OR gyroscope*
2. Search field topic for “shoulder”
3. Search field topic for “range-of-motion”
4. 1 AND 2 AND 3

Web of Science search strategy

1. Search field topic for kinect OR microsoft kinect OR motion tracking N6 technology OR digital inclinometer* OR inertial sensor* OR smartphone OR iphone OR accelerometer OR magnetometer OR magnetometer* OR gyroscope*
2. Search field topic for “shoulder”
3. Search field topic for “range-of-motion”
4. 1 AND 2 AND 3

Proquest Science and Technology search strategy

1. Search field anywhere expect full text for kinect OR microsoft Kinect OR digital inclinometer* OR inertial sensor* OR smartphone OR iphone OR accelerometer OR magnetometer OR magnetometer* OR gyroscope*
2. Search field anywhere expect full text for “shoulder”
3. Search field anywhere expect full text for “range-of-motion”
4. 1 AND 2 AND 3

Scopus search strategy

1. Search all fields for kinect OR microsoft Kinect OR digital inclinometer* OR inertial sensor* OR smartphone OR iphone OR accelerometer OR magnetometer OR magnetometer* OR gyroscope*
2. Search all fields for “shoulder”
3. Search all fields for “range of motion”
4. 1 AND 2 AND 3

Pubmed search strategy

1. Search all fields for "kinect" OR "microsoft kinect" OR ("motion tracking" N6 technology) OR digital inclinometer OR inertial sensor OR smartphone OR iphone OR accelerometer OR magnetometer OR gyroscope
2. Search all fields for “shoulder”
3. Search all fields for “range of motion”
4. 1 AND 2 AND 3
